# Effects Produced upon the Blood of Syphilitic Patients, by Treatment with Mercury

**Published:** 1876-03

**Authors:** 


### Effects Produced upon the Blood of Syphilitic Patients by Treatment with Mercury.

A paper upon the above subject was read at the meeting of the New York County Medical Society by Dr. E. L. Keyes. The following summary is taken from the *Record*. The paper may be found in full in the January nnmber of the *American Journal of Medical Sciences:*

With regard to the value of mercury in the treatment of syphilis,

Dr. Keyes assumes the following position: that syphilis is most surely controlled and most often cured by the unremitting use of small doses of mercury, just enough to restrain the symptoms without producing any conscious physiological effects, and continued for not less than two years. The drug may be pushed if symptoms seem to demand it, is often continued more than two years, and may be assisted by iodlde of potassium. The opinion was expressed, that when a syphilitic patient in fair health is treated in this manner from the period of his first eruption, he may be so cured that he will have but one general eruption, and no serious lesion subsequently; that the health of such a person will be as good after as before such treatment, and that the exceptions to the rule are not numerous. At this point reference was made to the statement of Liègeois, namely, that small doses of corrosive sublimate increase the weight of healthy men or animals. This statement has been sustained by the clinical observation of Dr. Keyes.

Reference was also made to the results obtained by Ricord and Grassi in the examination of the blood of syphilitic patients while they were being treated with mercury. The clinical experience of Dr. Keyes had seemed to contradict the result obtained by observers. Wilbouchewitch, in an article published in 1874, gave us his conclusions with regard to the effects produced upon the blood in the early days of syphilis by treatment with mercury. But Dr. Kyes was of the opinion that this observer had formulated his treatment for syphilis upon an erroneous interpretation of facts, and proceeded to show how his conclusions were worthless. Again, reference was made to the report of Dr. J. Hughes Bennett, in which the fact was stated, although no note made of it, that dogs increased in weight while taking small doses of mercury. In the light of the observations made by the gentlemen mentioned, Dr. Keyes began a series of experiments which extended over a period of six months, and were made upon persons in health, hospital patients, and patients in private practice. The blood was taken from twenty-seven individuals, of which six were apparently sound and twenty were syphilitic. Only three hospital cases were used, and in them the count was made chiefly for comparison. The blood was counted 101 times, and from five to ten counts made each time. The instrument used is called a *Hématimete.* All the methods of actually counting the blood cells, to which reference has been made by writers upon this subject, whether for red or white cells, have had the defect of not accurately establishing the count for a given volume of blood. The first recorded instance where accuracy was aimed at was reported by Cramer, but his process was a rough one. Reference was made to the iustruments which had been used, and a description of the instrument employed in his own observations. The fluid used to dilute the blood for counting was made as follows: Take urine, neutral, slightly phosphatic, about 1020 sp. gr., and make of it a saturated solution with borax. Filter, and there is left a fluid with sp. gr. about 1030, which is to be diluted with water until reduced to sp. gr. 1020. The following was regarded as a better fluid, but the corrosive sublimate may render it objectionable: Take of urine, neutral or slightly alkaline, sp. gr. 1020, filter, and add gr. v. of corrosive sublimate to the ounce, subsequently decant, and reduce with water to sp. g. 1020.

In the labor of the experiments Dr. Keyes was assisted by Dr. A. L. Stimson, and observations relating to the following points were noted :

1. *Average of red blood-corpuscles in* 1 *cubic mm. of blood of the healthy adult male.*—A high average is 5 millions. Anæmia rarely goes below 3 millions, and in five instances the count reached above 6 millions.

2. *Effect of small doses of mercury upon the blood early in syphilis.*—In all the cases counted, the number of red blood-corpuscles increased under the iufluence of mercury, good hygiene and tonics.

3. *Effect of the long-continued use of small doses of mercury upon the blood in syphilis.*—There were three cases; the drug was administered respectively, eleven, six and eighteen months; the blood-count was above the healthy average, and clinically they were all in excellent health.

4. *Effect of mercury in excess upon the blood in syphilis.*--In this, the only case in which salivation had been present (produced for special reasons), the count showed a loss of one million, which was attributed to the excessive use of mercury.

5. *Effect of mercury combined with iodides upon the blood in syphilis.*—In this list it would be fair to expect frequent exceptions to the rule, that mercury increases the number of red corpuscles in the blood of syphilitic patients, because so many who need prolonged treatment late in the disease become more or less cachectic and depreciated in general health, but in only two out of the nine cases under this head did the average count fall below the normal standard, and this among patients who had had syphilis for a long time.

6. *Effect of mercury upon the blood in syphilis in hospital cases.*—There were three cases. One entered salivated, and his count increased after he began to eat. One showed a wretched count, was debiliated by disease and hospitalism, but improved under the influence of good hygiene and tonics, and it was believed that mercury also helped him.

7. *Effect of small doses of mercury upon the blood of individuals not syphilitic.*—The observations showed an increase in the count. In testing the effect of small doses of mercury, Dr. Keyes used the protiodide in granules, gr. 1-5 each (the preparation more commonly employed by him in the treatment of syphilis); and, as every persons capacity for the drug varies, one granule was administered after each meal, and increased by one granule each day (not dose) on every fourth day, until some evidence of irritation was developed, when the dose was at once reduced to one-half, and there continued at the option of the doctor. The conclusions arrived at have in the main been given. The following may be added to make the report more complete:

1. Mercury decreases the number of the red cells when given in excess, especially in hospital cases.

2. Syphilis diminishes the number of red cells below the healthy standard.

3. Mercury in small doses continued for a short or long period in syphilis, given alone or with iodide of potassium, increases the number of red blood-corpuscles, and maintains a high standard of the same.

4. Mercury in small doses acts as a tonic upon healthy animals, increasing their weight. In larger doses it is debiliating or fatal.

5. Mercury in small doses is a tonic (for a time, at least) for individuals in fair health, not syphilitic. In such individuals it increases the number of the red blood-corpuscles.

The paper being open for discussion, Dr. Bumstead remarked that he regarded the results reported by Dr. Keyes as being of very great importance, and complimented the zeal with which the work had be pursued. The only criticism which he would venture was, whether the effect produced by mercury upon a healthy individual can be taken as a standard of the effect which will be produced by mercury upon an individual suffering from syphilis.

With reference to the results which follow a long-continued use of mercury in the treatment of syphilis, his experience had differed from that of Dr. Keyes.

Formerly he had treated syphilis according to the plan of administering mercurials for six months, perhaps a year, and following with iodide of potassium, which was to be continued for two or three months. The results obtained by this plan of treatment had, in his hands, proved very unsatisfactory.

Latterly, the plan of treatment which he has more frequently adopted consist in the vigorous use of mercurials for limited periods of time, and then the allowance of an interval, during which no remedy, except it may be a simple tonic, is taken. After a certain length of time, variable somewhat in different cases, a return is made to the mercury, when it is again pushed with considerable vigor. Dr. Bumstead preferred this course to the prolonged use of mercurial in small doses, as recommended by Dr. Keyes, and also by Jonathan Hutchinson, of London. With regard to the excessive use of mercury in the treatment of syphilis, such cases are rarely seen at the present time.

